# Nonattendance in pediatric pulmonary clinics: an ambulatory survey

**DOI:** 10.1186/1471-2466-9-12

**Published:** 2009-04-14

**Authors:** Aviv D Goldbart, Jacob Dreiher, Daniel A Vardy, Soliman Alkrinawi, Arnon D Cohen

**Affiliations:** 1 Department of Pediatrics, Soroka University Medical Center, Beer-Sheva, Israel; 2 Clalit Health Services, Tel Aviv, Israel; 3 Siaal Research Center for Family Medicine and Primary Care, Faculty of Health Sciences, Ben-Gurion University, Beer-Sheva, Israel; 4 Leumit Health Fund, Tel Aviv, Israel

## Abstract

**Background:**

Nonattendance for scheduled appointments disturbs the effective management of pediatric pulmonary clinics. We hypothesized that the reasons for non-attendance and the necessary solutions might be different in pediatric pulmonary medicine than in other pediatric fields. We therefore investigated the factors associated with nonattendance this field in order to devise a corrective strategy.

**Methods:**

The effect of age, gender, ethnic origin, waiting time for an appointment and the timing of appointments during the day on nonattendance proportion were assessed. Chi-square tests were used to analyze statistically significant differences of categorical variables. Logistic regression models were used for multivariate analysis.

**Results:**

A total of 1190 pediatric pulmonology clinic visits in a 21 month period were included in the study. The overall proportion of nonattendance was 30.6%. Nonattendance was 23.8% when there was a short waiting time for an appointment (1–7 days) and 36.3% when there was a long waiting time (8 days and above) (p-value < 0.001). Nonattendance was 28.7% between 8 a.m. to 3 p.m. and 37.5% after 3 p.m. (p = 0.007). Jewish rural patients had 15.4% nonattendance, Jewish urban patients had 31.2% nonattendance and Bedouin patients had 32.9% nonattendance (p < 0.004). Age and gender were not significantly associated with nonattendance proportions. A multivariate logistic regression model demonstrated that the waiting time for an appointment, time of the day, and the patients' origin was significantly associated with nonattendance.

**Conclusion:**

The factors associated with nonattendance in pediatric pulmonary clinics include the length of waiting time for an appointment, the hour of the appointment within the day and the origin of the patient.

## Background

Nonattendance (failure to attend clinic appointments) is a universal problem in the management of pediatric clinics. In general, nonattendance rates in the United States (US) range from 5 to 55%, whereas United Kingdom (UK) figures range from 3 to 12%. In Denmark, a very low rate (4%) has been reported [1]. Nonattendance leads to a wasteful use of manpower and increased clinic costs [1-3] Nonattendance has both social and financial costs. Social costs include unused resources, such as personnel time, equipment and clinic capacity. Another issue is the possible misuse of patients' time, including the inability to execute their jobs and loss of future production. Financial costs include the providers' lost of income because of lower reimbursement than that achievable without nonattendance, due to the use of fee-for-service or per case schemes, where a no-show appointment is not reimbursed by a third party payer. The amount of evidence regarding costs of nonattendance is modest, as few studies have explored the costs of nonattendance. Many of these have been unsophisticated or even misleading [1]. The determinants of nonattendance include disease-related factors (e.g. acute vs. chronic disease), patient-related factors (e.g. patients forgetting their appointment, transportation difficulties, inability of the child or his parent to leave school or work, inadequate communication between the clinic and patients, including mix up over the date or time or unexpected events), demographic and socioeconomic factors, office accessibility and factors related to the health care provider [4-10].

In previous studies we assessed the factors that determine nonattendance in adult dermatology clinics [11] and pediatric otolaryngology clinics [12]. It was observed that the waiting time for the appointment and the timing of appointment during the day were significant determinants of nonattendance [11,12]. However, a literature search failed to reveal studies on nonattendance in pediatric pulmonary clinics, except a single study describing nonattendance at pediatric asthma clinics [13]. The explanations for non-attendance of patients and their families and the measured that are taken to solve this phenomenon might differ between specialties, according to the nature of potential complaints (e.g. seasonal phenomena, recurrent vs. chronic illnesses, and so on). We expected that the reasons for this problem and the derived corrective strategies might be different in pediatric pulmonary clinics. In the current study we therefore investigated the factors that determine nonattendance in children attending a pediatric pulmonary clinic. We hypothesized that a longer waiting time, belonging to an ethnic minority (Arab Bedouins) and certain age groups (e.g. adolescents) might be associated with a higher rate of nonattendance.

## Methods

The study was conducted in the pediatric pulmonary clinics of the southern district of Clalit Health Services, the largest health-maintaining organization in Israel. All first-time visitors to the clinic under the age of 18 years during the study period were included in the study. Visits included in the study were performed during the regular working hours of the clinics (8 a.m. – 7 p.m., Sunday through Thursday, and 8 a.m. – 12 p.m. on Friday). Pulmonology clinics were held on Sundays, Mondays and Thursdays. All subjects paid for their office visit according to the National Health Insurance Law of 1995, a uniform co-payment fee of 12 NIS (approximately 3 US $) which was valid for all subsequent visits within a calendar quartile.

Visits to the pediatric pulmonary clinics were obtained from a computer-generated list of all scheduled appointments from April 1^st ^2003 to December 31^st^, 2004. The list contained the patient's age, gender, origin, visit hour and waiting time for an appointment for all new appointments in the clinic. Patients were not required to pre-authorize the appointment and did not receive a reminder of the scheduled appointment, such as a telephone call or a reminder letter. The study was exempt from submission to the local ethics committee as there were no identifiable data which was used in the study, after the initial data mining process was finished.

### Statistical Analysis

Continuous variables are shown as means and stadard deviations. Categorical variables are described as frequencies. Chi-square tests were used to analyze statistically significant differences of categorical variables. Logistic regression models were used for multivariate analyses. Two-tailed p values less than 0.05 were considered statistically significant.

## Results

A total of 1,190 pediatric visits were included in the study during the 21-months period. There were 418 females (35.1%) and 772 males (64.9%) patients. The mean age was 6.3 ± 5.3 years. Other descriptive characteristics are listed in Table 1.

**Table 1:** Descriptive characteristics of the study population. (n = 1,190)

Variable	N (%) or Mean ± Standard deviation
Age (years):	
Mean	6.3 ± 5.3
Median	5
Range	0 – 18
< 1 year	231 (19.4%)
1–5 years	438 (36.9%)
6–11 years	276 (23.2%)
12–15 years	170 (14.3%)
16–18 years	73 (6.1%)
Male Gender	772 (64.9%)
Origin:	
Rural Jewish	91 (7.7%)
Urban Jewish	667 (56.1%)
Bedouin	432 (36.3%)
Season:	
Winter	282 (23.7%)
Spring	337 (28.3%)
Summer	319 (26.8%)
Fall	252 (21.2%)
Day of the week:	
Sunday	245 (20.6%)
Monday	585 (49.6%)
Thursday	360 (30.3%)
Hour of the day:	
8 AM to 3 PM	937 (80.4%)
3 PM to 4 PM	59 (5.2%)
4 PM to 7 PM	170 (14.9%)

Waiting time (days):	
Mean	11.2 ± 8.8
Median	9
Range	0 – 68
< 7 days	428 (36.0%)
7–14 days	380 (31.9%)
> 14 days	382 (32.1%)

The overall proportion of nonattendance at the pediatric pulmonology clinic was 30.7%. Nonattendance proportion was slightly higher among males, and in certain age groups (1–5 years old and over 12 years old), but the differences were not statistically significant. Jewish rural patients had 15.4% nonattendance, Jewish urban patients had 31.2% nonattendance, and Bedouin patients had 32.9% nonattendance (p = 0.004) (Table 2)

**Table 2:** Nonattendance proportions in different subgroups (n = 1,190)

Variable	Nonattendance – N (%)	P value
Overall	365 (30.7%)	-
Age:		
< 1 year	61 (26.4%)	0.143
1–5 years	143 (32.6%)	
6–11 years	74 (26.8%)	
12–15 years	60 (35.3%)	
16–18 years	25 (34.2%)	
Gender:		
Male	244 (31.6%)	0.300
Female	120 (28.7%)	
Origin:		
Rural Jewish	14 (15.4%)	0.004
Urban Jewish	208 (31.2%)	
Bedouin	142 (32.9%)	
Season:		
Winter	81 (28.7%)	0.025 (Winter & summer vs. spring & fall)
Spring	114 (33.8%)	
Summer	85 (26.6%)	
Fall	84 (33.3%)	
Day of the week:		
Sunday	91 (37.1%)	0.037
Monday	173 (29.6%)	
Thursday	100 (27.8%)	
Hour of the day:		
8 AM to 3 PM	269 (28.7%)	0.015
3 PM to 4 PM	26 (44.1%)	
4 PM to 7 PM	60 (35.3%)	
Waiting time:		
< 7 days	100 (23.4%)	< 0.001
7–14 days	119 (31.3%)	
> 14 days	145 (38.0%)	

Nonattendance was higher during the spring and fall, compared with the summer and winter (p = 0.025). Nonattendance was higher on Sundays than on other days. The proportions of nonattendance varied according to the hour of the day from 28.7% between 8 a.m. to 3 p.m. to 44.1% between 3 p.m. and 4 p.m. and 35.3% after 4 p.m. (p = 0.015) (Table 2). Nonattendance increased as the waiting time for an appointment increased, from 23.4% among those waiting for less than 7 days, to 31.3% and 38.0% when there was a longer waiting time (7–13 and 14 days and above, respectively) (p < 0.001).

A multivariate logistic regression model demonstrated that the waiting time for an appointment, the hour of the day and the origin of the patients were significantly associated with nonattendance (Table 3). Other factors suspected to be associated with nonattendance based on the univariate analysis (season, age group, day of the week) were not found to be statistically significant covariates in the multivariate model.

**Table 3:** Logistic regression model for factors associated with clinic nonattendance in pediatric pulmonology patients.

Variable	OR	95% CI	P value
Male gender (vs. female)	0.86	0.65–1.13	0.275
Age (per year)	1.02	0.99–1.05	0.245
Origin:			
Rural Jewish (reference)	1.00	-	-
Urban Jewish	3.39	1.80–6.37	< 0.001
Bedouin	2.95	1.56–5.57	0.001
Hour:			
8 AM to 3 PM (reference)	1.00	-	-
3 PM to 4 PM	2.19	1.26–3.81	0.005
4 PM to 7 PM	1.45	1.01–2.08	0.039
Waiting time:			
< 7 days (reference)	1.00	-	-
7–13 days	1.65	1.20–2.28	0.002
≥ 14 days	2.18	1.57–3.01	< 0.001

OR = odds ratio, CI = confidence interval.

## Discussion

Clinic nonattendance is a difficulty that affects all medical specialties. Failure of patients to show for scheduled appointments wastes limited medical resources and prolongs waiting time for appointments. In particular, high proportions of nonattendance consumes private and institutional resources as the majority of clinic expenses are fixed, whereas costs that vary with patient attendance are small [1-3].

In the current study, we investigated the factors for nonattendance in pediatric pulmonary clinics. The overall nonattendance proportion was 30.7%. The waiting time for an appointment, the hour of the day and the origin of the patient were significantly associated with nonattendance. The proportion of nonattendance was higher for visits with a long waiting time and those taking place after 3 p.m (and especially between 3 p.m. and 4 p.m.). A Jewish rural origin was inversely associated with the risk of nonattendance.

The determinants of nonattendance include disease related, patient-related and health care provider-related factors [4-10]. Disease-related factors include the length of time the patient has been afflicted with the disease and the type of illness (the presence of significant distress and symptoms, possibility of spontaneous resolution of symptoms) [6]. Patient-related factors include age, ethnicity, gender, health-belief factors, social deprivation, an urban setting, and travel time, as well as days of the week or poor weather (extremes of mean daily temperatures, especially during winter). [1,6] Health care provider related factors include the insurance type (HMO-based program having higher nonattendance than a Medicare-based program), source of referral (e.g. from the treating physician or from the emergency department), type of physician (resident vs. staff physicians) and the waiting time for an appointment [4,6]. Penneys et al [14], who described nonattendance in 4876 dermatological patients, observed that the payer type was significantly associated with nonattendance; nonattendance proportion was 26% in patients with public insurance as compared with 13% in patients with private insurance. Hon et al [15,16] found that referrals from the emergency department to dermatology clinics were associated with higher proportions of nonattendance as compared with referrals from private physicians. Dickey et al [17] found that patients who waited less than 2 months for an appointment for a neurologist had a nonattendance proportion of 17%, as compared with 32% if the waiting period exceeded 2 months In a study of pediatric asthma clinics [13], factors associated with non attendance were the type of insurance and seasonality: a higher rate of nonattendance was associated with Medicaid insurance and visits scheduled in September–December.

The literature on nonattendance is diverse, and the reasons for nonattendance seem to vary between children and adults, as well as between ethnic groups and different medical conditions. Therefore, in the present study we examined factors associated with nonattendance among children visiting a pulmonology clinic.

We observed that the origin of patients was a significant factor for clinic nonattendance. Jewish rural patients had a lower nonattendance proportion (15.4%) than Jewish urban (31.2% and Bedouin (32.9%) nonattendance proportions. The Bedouins in the Negev include about 130,000 people. They are an ethnic, religious, and cultural minority. Their religion is Islam and their language is Arabic. The Bedouins are a population in transition from a nomadic to a settled and urban form of life. Currently, approximately half of the Bedouin population live in urban settlements and the other half live in the rural settlements. Many of the Bedouins are in a lower socioeconomic level with high unemployment rates. Many families live on social security income. The medical services offered by the health authorities are not fully used due to language, cultural, and economic difficulties and also due to the geographical scattering. The transition from a traditional to a Western lifestyle, characterized by changes in dietary habits and a reduction in physical activity is associated with substantial changes in morbidity patterns such as a considerable increase in diabetes prevalence and childhood injuries [18,19].

The observation of high nonattendance proportions of Bedouin patients in pediatric pulmonary clinics is compatible with the socio-demographic characteristics of the Bedouin population as described above. Social deprivation as risk factor for nonattendance can be explained by the fact that a lower level of education, a lower level of health literacy, loss of wages, less flexible work schedules, and limited access to transportation would be more relevant to one in a lower socioeconomic group [6,20]. In a study of nonattendance at an orthodontic clinic, utilizing reminder letters, social deprivation was associated with a higher risk of nonattendance (Odds ratio: 2.7, 95% confidence interval: 1.1 to 6.5), even after controlling for the use of a reminder letter [5]. The behavior of providers, including difficulties to communicate in Arabic, inadequate sensitivity to cultural issues, and the difficulty to correctly assess the level of health literacy [20] could potentially contribute to the higher nonattendance rate seen among Bedouins. The high nonattendance proportion in Jewish urban patients is unexpected, in particular, in view of the low nonattendance proportions in Jewish rural patients who also experience problems with accessibility. Another possible explanation is that Jewish non-rural parents have a greater access to the clinics due to geographic proximity. An urban setting has been described as risk factor for nonattendance in other studies [6].

There are several limitations to this study. We did not collect data about the diagnosis or presenting problem, referral source or severity of the disease. No indicators of socioeconomic status (income, education, family composition and size) were available and this lack of data may confound the conclusions. Therefore, potential recommendations are limited.

Given that nonattendance is such a significant problem, various interventions have been suggested to deal with it. These can be grouped into three main categories: reminders (reminder letters, telephone reminders, short message service [SMS]), reducing perceived barriers (information given to patients before appointments, regarding the reasons for the appointment, what patients may expect when they arrive and how the clinic is organized), and increasing motivation, by "punishing" non-attendees by discharging them from the waiting list or by assigning them to the bottom of the waiting list, charging non-attendees a monetary fine, positive financial incentives for compliant patients (cash reward, gifts, vouchers, or lottery tickets) and contracting – a process where a formal agreement is made with patients to attend future appointments and to agree to treatment plans. While some suggest that charging a fine may punish certain types of patients – especially fragile and low-resource patients, a recent survey among general practitioners in the UK revealed that two thirds believed that non-attendees should be fined [1,2,5,9]. Another possible solution is overbooking, in order to counteract the problem of unused resources. Overbooking results in patients waiting longer for their appointment. This type of intervention is advantageous to the provider, since the costs associated with nonattendance are shifted to the patients. In evaluating all these interventions, one should ask whether they are effective at reducing nonattendance and what is the magnitude of costs associated with the suggested intervention [1]. Managed overbooking, which includes overbooking in certain subgroups of patients, providers, and time-slots, could be a potential solution. For example, in the clinic described in the present study, overbooking during certain hours (3 p.m. to 4 p.m.), among certain populations (urban Jewish and Bedouins), etc could compensate for the higher rate of nonattendance. Alternatively, focusing reminders on populations prone to fail to attend could be a cost-effective way to decrease nonattendance. Another possible solution (often hard to implement) is reducing the waiting time for the appointment. Based on our findings, it seems that the optimal time from referral to appointment should be one week.

## Conclusion

In summary, in the current study we found that the waiting time for an appointment, the hour of day and the origin of the patient are important factors that determine nonattendance in pediatric pulmonary clinics. To increase the efficiency of pediatric pulmonary clinics various interventions, such as managed overbooking lists should be considered.

## Competing interests

The authors declare that they have no competing interests.

## Authors' contributions

ADG conceived of the study, participated in the design of the study, drafted the manuscript and helped in the interpretation of results. JD participated in the design of the study, performed the statistical analysis, helped in the interpretation of results and drafted the manuscript. DAV participated in the design of the study, helped in the interpretation of results and in drafting the manuscript. SA participated in the design of the study, and helped in the interpretation of results and in drafting the manuscript. ADC conceived of the study, participated in the design of the study and the collection of data, helped in performing the statistical analysis, and drafted the manuscript. All authors read and approved the final manuscript

## Pre-publication history

The pre-publication history for this paper can be accessed here:


